# Toward a phenomenology of taking care

**DOI:** 10.1080/17482631.2022.2045671

**Published:** 2022-03-15

**Authors:** Christophe Coupé, Magali Ollagnier-Beldame

**Affiliations:** a Department of Linguistics, The University of Hong Kong, Hong Kong, China; b Laboratoire Ddl, Cnrs, University of Lyon, Lyon, France; c Laboratoire Icar UMR5191, Cnrs – University of Lyon – Ens Lyon, Lyon, France

**Keywords:** Subjective experience, caregiver – patient relationship, micro-phenomenology, explicitation interview, finalized activity

## Abstract

**Context and Purpose:**

From nurses to dentists and doctors, caregivers undergo significant initial and life-long training. This training, however, rarely addresses the subjective side of their practice, especially the lived experience of caregiving. Better understanding this experience can nevertheless help to build fruitful relationships with patients. We focus on what it is like to take care of someone else and attempt to outline an encompassing “phenomenology of care”.

**Methods:**

We investigate the lived experience of caregivers during their first meeting with a patient. We rely on micro-phenomenological interviews, which offer fine-grained, first-person descriptions of someone’s holistic experience in a given situation.

**Results:**

We show how the subjective experience of meeting a new patient can be structured with i) categories of micro-experiential acts (gathering information, assessing and performing actions), ii) the scopes of these acts, which involve inner and outer perceptions, various elaborations, regulations and interventions and iii) a range of experiential modalities.

**Conclusion:**

We highlight the richness of lived experience, and what all caregivers intimately share beyond the frame of their respective professions and practices. We discuss our results in terms of methodology, finalized and productive activities, pre-reflective aspects, and reflexive practice.

## Introduction

The ongoing COVID-19 pandemic has shed a crude light on the need of strong health care systems and what may happen when they are institutionally, and more broadly socially, neglected. Some (Legrand, 2020; Legros, 2020) have noticed in particular how the current situation revitalizes the theory and ethics of care promoted by Tronto (2009). This author has indeed emphasized the growing importance of paying attention to and taking care of others, and of establishing care and well-being as pillars of our societies.

In Western countries (Europe, Northern America), caregivers play a key societal role. This role can, however, be downplayed in the sense that caregiving is actually so present and internalized that it becomes invisible. Indeed, in these countries, constant health care starts at the first moments of life for most individuals. This is particularly the case in France where our study took place. The COVID 19 pandemic has clearly revealed that caregivers were impacted by increased workload, overexposure to infectious risk, reorganization of care and routine changes. These challenges have been amplified by isolation and loss of social support, and have overall fed a feeling of one’s role being minimized (Pappa et al., 2020). This has led to psycho-traumatic, anxiety, and depression symptoms (Vignaud & Prieto, 2020), sleep disorders (Bertrand et al., 2021), and burnout (Franc-Guimond & Hogues, 2021).

Nurses, doctors, physiotherapists, psychotherapists etc. weave a dense network of skills and practices to serve the population on a daily basis, even if those assets may not be properly recognized. In such a framework, trying to better understand what is at play for caregivers’ daily practices is a meaningful endeavour. What is it like to be a caregiver and to take care of someone else? What is the related subjective experience, and how does actual practice differ from prescriptions? What may constitute a phenomenology of care, beyond the specificities of different professions?

### Relevance of the caregiver—patient relationship in care

In many situations of care, the establishment and maintenance of the intersubjective relationship between the caregiver and the patient are crucial issues for effective patient support. As a counterpoint to the biomedical model that was dominant until the 1970s, the biopsychosocial model developed in 1977 by Engel (Siksou 2008) emphasized for instance, that: i) the doctor-patient relationship influences the medical outcome, if only by influencing the adherence to treatment, ii) the doctor’s effectiveness is linked to the fact that her/his personality is an instrument of therapeutic change, and iii) the patient’s subjective experience deserves attention as much as the disease (*ibid*.). Engel thus connected biological, psychological, and social factors into a complex causal system. In this way, he contributed to countering the dehumanization of medicine and the infantilization of patients. This perspective has recently been reinforced, for instance, with the development of therapeutic education for patients (Assal, Golay & Visser, 1995; Vargas-Schaffer & Cogan, 2014) and the growing role of “expert patients” engaged in therapeutic education (Cuvelier, 2018).

Professions in healthcare differ in the precise place occupied by the caregiver-patient relationship. While a trauma surgeon in emergency situations may not have much chance to interact significantly with those s/he treats, nurses working at the bedside of patients provide far more than biomedical interventions and assessments of their health status. Further, while the intersubjective relationship may sometimes be seen as supportive yet not the most central element of care, it is a prerequisite in psychotherapeutic approaches: the so-called “therapeutic alliance” (Macewan, 2008; Sexton et al., 2005) or “therapeutic relationship” (Ardito & Rabellino, 2011), has been highlighted in a number of studies, with focus for instance, on the “affective attunement” (Stern, 1983, 2004). It has been debated whether the therapeutic outcome depends on a positive therapeutic alliance early in treatment (Barber et al., 2000).

The modality and temporal unfolding of the relationship also differ across professions: in some domains like nursing, medicine or physiotherapy, interactions are in particular mediated by touch, a form of connection which intimacy needs to be carefully addressed (Kelly et al., 2018). In the field of oncology, the moment when the patient is told about her/his illness has been carefully considered (Beach et al., 2005; Couitchéré et al., 2019). Indeed, how the oncologist handles the announcement can have lasting consequences on the patient’s attitude towards her/his affliction. More generally, how first meetings contribute to the therapeutic alliance and the success of the treatment has been emphasized (McAllister et al., 2004).

Beyond the previous differences across professions in healthcare, one can also look for similarities, i.e., what may be considered at the core of healthcare. One can mention here the metasynthesis of phenomenographic articles on understandings of work among healthcare professionals conducted by Roïng, Röing et al. (2018). These authors highlight a hierarchy of ways of understanding which, from a limited to a more comprehensive view on patients and on their needs, unfolds as:

- Meeting the challenges of being a healthcare provider;
- Providing biomedical care to patients;
- Caring for patients as vulnerable individuals;
- Promoting patient independence and responsibility for own care;
- Ensuring holistic safe and effective healthcare services.

Another point most healthcare professions, if not all, have in common is the asymmetry of role and situation between the caregiver and the patient. This is true in particular during the first meeting, and despite increasing consideration paid to the latter by the former. Both actors are ascribed to specific roles, which can shape their behaviour: while the caregiver is often considered as the depositary of medical knowledge and therefore occupies the higher grounds, the patient is usually both devoid of this knowledge and weakened by what led her/him to meet the practitioner. However, both the caregiver and the patient are more than what social rules and the situation constraint them to be. They retain who they are as persons, which contrary to what precedes defines a more symmetrical interaction. This may not, nevertheless, easily surface during the interaction, since medical training heavily insists on the role the caregiver must adopt, and how s/he must leave aside personal emotions in front of the patient.

Across a number of healthcare professions, being able to assess patients’ personality and role may prove precious for the caregiver during a first meeting. Even outside of psychotherapy, where this is a central requirement, and unless biomedical aspects occupy the whole space like in surgery, a proper assessment may help to better interact with new patients, and to craft more genuine and effective therapeutic relationships. However, this is not an easy task, first because meeting new patients is repetitive and usual. Although the very first meetings with patients may be noticeable moments for a new practitioner, habits soon step in and decrease one’s attention to the process. Second, while they occur repetitively, first meetings engage at the personal level and caregivers may avoid behaviours creating discomfort or awkwardness. Third, caregivers, to the exception of psychotherapists, are often trained neither to fully pay attention to the features of this crucial moment on the spot, nor to look back at it reflectively and reflexively.^1^ It therefore often constitutes a blind spot. One may thus wonder whether the ethical imperative “never the first time on a patient”—increasingly influential among professionals, and encouraging the use of dummies and simulated environments (Cuvelier, 2018)—should be replaced by the alternative principle: “always the first time with a patient” (Lechopier, 2020). If so, what particular skills do caregivers need to mobilize to “succeed” in their first meetings?

### Experience in care

The majority of caregivers receive both theoretical and practical initial instruction (internships, simulations, etc.), and many of them undergo further training throughout their careers. More often than not, little room is offered to lived experience in situations of care, whether it is the patient’s experience or the caregiver’s. Anthropologist Byron Good looked at how caregivers accommodate (or not) the patient’s experience. His work on the construction of health professionals shows how medical students at the university develop their listening style—especially regarding patients’ voices and illness narratives—and their attitudes over the years of their curriculum (Good, 1994). For instance, Good studied the linguistic qualities of illness narratives. He showed that the context of the telling may influence the way the story is structured. This paves the way for an analysis of the complex relation between the patient’s experience and her/his narrative representations of illness. More broadly, accounting for the lived experience^2^—both the patient’s and the caregiver’s—and studying it in-depth offer additional perspectives to those of studies that rely only on observable data and/or non-experiential verbal reports. For instance, in a study about the lived experience of first meetings between caregivers and patients, Ollagnier-Beldame and Cazemajou (2019) highlighted a type of judgement that appears to be central in the intersubjective skills of healthcare workers, yet has hardly been investigated: intellectual judgements (Burloud, 1927), also called embodied judgements by the authors. They are part of a tacit category of judgment, linked to intuitive thinking and a specific mode of awareness (Messer, 1906) or affective state. Usually expressed by “I feel” (e.g., *“I feel something strange, I feel that something is happening to him, which is a bit strange”*), they do not convey only a sensory feeling but are directly related to the therapists’ pre-reflective^3^ inner organization and accumulated skills.

The previous results suggest how the activity of meeting someone and the corresponding subjective experience may take shape and unfold over time. They also promote healthcare workers’ reflexive practice. This reflexive dimension is at the heart of the analysis of practices (Henry, 2019) and can be an essential component of an ethics of care. In this way, the access to lived experience, its precise description and its reflexive analysis are a source of learning which has the potential to transform caregivers’ representations and attitudes towards future patients as potential partners for better care. The section below introduces an epistemology that allows such a study of subjective experience.

### First-person perspective and epistemology

Studying lived experience “from within” requires a specific epistemology. Phenomenological approaches make a difference between perspectives in first, second and third person by separating the perspective of the subject who lives the experience from the perspective of another subject, such as the researcher (Depraz et al., 2003). A first-person epistemology considers subjectivity as it is experienced by the subject herself/himself (Depraz, 2014; Shear & Varela, 1999), and embraces both the perspectives in first (the “I” speaking is direct, immediate) and second person (the “I” speaking is mediated by another person, e.g., an interviewer). The point is to enable access to the experience as it is lived by the subject, i.e., from her/his own unique perspective.

The first-person epistemology is opposed to the third-person epistemology in which subjectivity and lived experience are generally viewed as epiphenomena or beyond the reach of science (Vermersch, 2000a), and observable behaviours are examined according to predefined categories. This relates to a deep-seated lack of confidence in the validity of the data drawn from introspection (Nisbett & Wilson, 1977), although such data have been given renewed legitimacy (Hurlburt & Heavey, 2006; Petitmengin et al., 2013). It is important to bear in mind that the first-person epistemology is not an epistemology of immediacy since experience, while it may be lived and familiar, has a pre-reflective dimension. Knowing it in detail presupposes an objectification of one’s subjectivity—no easy task despite the apparent transparency of intimacy and familiarity. Thus, the first and second-person perspectives should not be confused with immediate donation, i.e., for the subject, a sudden, clear and distinct illumination (Vermersch, 2000b).

‘Indeed, being epistemically related to facts about oneself (“I”) is not a sufficient condition for first-person perspective taking: You can also have an objective, third-person view on your headache. […] What is needed is a difference not in terms of the epistemic object but, rather, in terms of epistemic access – even if it may turn out to be necessary to refer to specific epistemic objects in order to clarify what the specific kind of access is. The decisive point seems to be that there are certain features of oneself that do require a specific kind of epistemic access’ (Pauen, 2012, pp. 37–38).

This crucial question of the epistemic access to experience has led to the development of many experiential data collection methods (Gendlin, 1997; Giorgi, 2009; Hurlburt & Heavey, 2006; Petitmengin, 2001; Vermersch, 1994). They offer different possibilities of “survey” and experiential description, depending on the type of experience and research goals. To us, the method both most complete and most suitable for describing action is the micro-phenomenological interview, also called explicitation interview, to which the first section in “Materials and methods” is dedicated. Many researches are based on it (Dieumegard et al., 2021; Ollagnier-Beldame & Cazemajou, 2019; Petitmengin, 2001; Petitmengin, 2006; Przyrembel & Singer, 2018), some of which study situations of care (Denis, 2016; Ollagnier-Beldame & Cazemajou, 2019).

## Materials and methods

### The micro-phenomenological interview as a data-collecting tool

To collect our data, we relied on the micro-phenomenological interview, also called explicitation interview. This technique was initially developed in the 1990s and later elaborated, especially in terms of analysis, by Pierre Vermersch (1994, 2012), Petitmengin (2006, 2019) and members of the GREX (Research Group in Explicitation). In contrast with other types of interviews, which focus on social representations (Moscovici, 2000), it is concerned with lived experience. Theoretically, it rests on several complementary contributions in philosophy, psychology, and phenomenology:

- Edmund Husserl (1933)’s phenomenology, which plays a central role with notions such as retentions (echoes of the immediate past in the present time) and protentions (the potential “future of the present” as it exists in the present time). They highlight how situatedness in time is a defining feature of one’s present-time subjective experience, and this outlines the importance of studying peculiar and specific moments (Vermersch, 2012), unless one is interested in classes of experiences and generalizations.

- Gusdorf (1951)’s theory of affective memory, which posits that lived experience is constantly and passively memorized;

- Piaget (1974)’s theory of consciousness, which emphasizes that part of one’s consciousness is implicit, a “consciousness in action” which may, however, be brought to conscious awareness and verbalized;
- Gendlin’s philosophy of the implicit (Gendlin, 1978) and focusing interview (Gendlin, 1997), which show how experiencing a situation is always embodied. This vague felt experience is known as “bodily felt sense” and contains meaning regarding the situation as a whole.

Following Husserl and the other aforementioned authors, Vermersch (2008) describes three modes of consciousness: i) a phenomenological unconscious (cf. Husserl), during which lived experience is passively memorized (cf. Gusdorf), before any intentional act; ii) a mode of reflective consciousness, which corresponds to what the subject is aware of; iii) in between, a pre-reflective mode of direct consciousness, or consciousness in action, which at a given moment includes all of one’s perceptions, sensations and feelings (cf. Gendlin) but is not readily accessible, though its content can eventually reach reflective consciousness through a process of reflection (cf. Piaget).

The micro-phenomenological interview can be described as a technique of guided retrospective introspection, which means that the interviewer (commonly named B), with carefully chosen questions, assists and guides the interviewee (named A) as s/he recalls and revisits a past experience. Under the interviewer’s guidance, the interviewee is led to suspend her/his judgement—a process known as the Husserlian *epoché* (Depraz et al., 2003). More specifically, according to Depraz, Varela and Vermersch:

‘one accomplishes the *epoché* in three principal phases:

A0: Suspending your “realist” prejudice that what appears to you is truly the state of the world; this is the only way you can change the way you pay attention to your own lived experience; in other words, you must break with the “natural attitude.”

A1: Redirecting your attention from the “exterior” to the “interior.”

A2: Letting-go or accepting your experience.’

(Depraz et al., 2003, p. 25)

The *epoché* is thus what allows the interviewee to access her/his past lived experience. Of paramount importance is the open and non-inductive nature of the questions, which results in a guidance that is attentional rather than orienting the content of the answers. Concretely, when exploring a perceptual episode, the initial question “what do you perceive?” may for instance, be more adapted than the question “what do you see?”, as it does not imply the perceptual modality that was then dominant for the interviewee. More generally, perlocutionary effects of the interventions are controlled as much as possible to prevent the possible induction of distorted or false memories (Schacter, 2001). The interviewer’s interventions also aim to induce and maintain the interviewee’s activity of introspection, with a process of letting go favouring the surfacing of the past experience. The slightly modified state of consciousness which favours a presence to oneself and an intimate contact with the past situation is called the “embodied posture of speech”. It is not spontaneous and can also be endangered, for instance, when recapitulations deviate from the interviewee’s precise words and descriptions. Careful listening is thus essential, as well as regular assessments of complementary verbal and non-verbal cues—unfocused eyes, the slowing down of speech, the use of the personal pronoun “I” rather than “we” etc.

Overall, the set of interventions is limited by several factors: first, the objectives of the interview, i.e., the nature of the information one wishes to collect; second, what is theoretically known of experience, and which corresponds to rather high-level generic processes (e.g., the model of action below); and third, what the interviewee tells the interviewer. Interventions thus mostly consist in recurrent question frames, with a limited set of verbs and temporal articulations, e.g., “[and then/just before/just after,] what do you feel/perceive/do/hear”, “[at that very moment/just before/ …] how do you know that?”, “how does it feel?”, “what do you do/pay attention to/feel [when you + verb of action]?” etc.

The aim of the micro-phenomenological interview is to collect detailed descriptions of a singular lived experience. These descriptions aim to be holistic in the sense that the various components of experience, from cognitive operations and physical actions to sensations and emotions, are considered and deemed of interest. What are sought are specific descriptions of what was experienced, and not generalizations derived from repeated situations and habits, nor opinions. This explains in particular why “why” questions are disregarded, as they tend to induce unwanted comments and opinions. Interviewers look for both fragmentation, which corresponds to the collection of the diachronic succession of microscopic experiential events that compose larger-scale experiences, and for qualitative expansion, which points at the various facets in synchrony of the experiential events. The pre-reflective aspects of the experience under study, i.e., the part of it that remained below the threshold of consciousness, are given special attention since they may contain useful information regarding what was lived and how it unfolded. This includes in particular the possible sources of errors made during an activity or implicit aspects of expert knowledge.

As it targets first-person descriptions, the micro-phenomenological interview differs from third-person methods, where data is collected from an external viewpoint, often considered to be objective. In micro-phenomenology, words are endowed with considerable trust, as they provide (the only) access to subjective experience. While it is not always possible to assess the extent of the semiotic transformation implied by the wording of a “wordless” experience, it makes sense to assumes that different choices of words point to subtle differences between experiences: the presence of a sense of agency in *“I feel that she is scared”* suggests a different experience from the absence of the same sense of agency in *“This feeling that she is scared, that’s what comes to me”*.

### Process of data collection for the study

A total of 13 caregivers from various care professions were interviewed: two speech therapists (both female), two family medicine practitioners (one female, one male), one dentist (male), one physiotherapist (female), three psychotherapists (two females, one male), four nurses (with various specializations, three females, one male). All participants were native French speakers. The interviews took place in the interviewee’s workplace or in a meeting room located in one of the authors’ research institutions. No strict control was enforced for variables such as the interviewee’s gender or the match or mismatch between the interviewer and interviewee’s genders.

This study follows the Helsinki Declaration (World Medical Association, 2013). The primary objective of our research was to generate new knowledge on care, without prevailing over the health, well-being, and rights of our participants. Along these lines, all the interviewees were experienced and practising health professionals.

With each of our interviewee, we took first ample time to i) introduce the aims, methods and institutional framework of our research, including data collection for the sole purpose of research and data anonymization, ii) inform the interviewee that s/he could stop the interview at any time for whatever reason, iii) explain the interviewee that s/he was entirely free to choose the first meeting s/he was most interested in, and to keep elements of this meeting for her/himself if s/he felt the need to, and iv) answer any possible question. We obtained full and explicit oral consent to participate in the study before starting the audio-recorded interviewing process. No information regarding the identity of the patients that had been met by the interviewees was ever collected, and no name was ever mentioned.

We chose not to ask for a written consent, as we feared this more formal approach could increase the asymmetry of the relation and lead the interviewee to watch her/his words more restrictively. Micro-phenomenological interviews indeed require a lot of trust, as the interviewee usually discloses intimate experience s/he is not used to share. Increased formality could also have led caregivers to act as professionals even in front of the interviewer.

Each interview lasted between 45 and 90 minutes. The total duration of the recordings was 14 hours, 7 minutes, and 42 seconds. 10 or the 13 recordings were fully transcribed. The 10 micro-interviews proper lasted together 552 minutes and 41 seconds, with a mean duration of 55 minutes and 16 seconds and a standard deviation of 14 minutes and 57 seconds. Their transcriptions, with added information about time and numbered interventions assigned to the interviewer or the interviewee, contained a total of 117,142 words, with a mean number of 11,714 words per transcription and a standard deviation of 3,286 words. Transcriptions are available upon request to the corresponding author.

Information on the educational and training background, the number of years of practice and the professional context of intervention (private/public practice, patients etc.) was also collected to provide a number of meta-data for later analysis.

Upon carefully reading the 11 transcriptions, 7 were considered for further analysis.

### Analytical approach to the interviews and transcriptions

Our analytical approach to experience is qualitative and faces the epistemological and methodological challenges of all qualitative approaches. These challenges relate to the bottom-up construction, organization and selection of relevant possible interpretations of one’s raw material (Saldaña, 2011) As described below, our method rests on a time-tested iterative approach, in order to extract meaningful descriptive categories of experience.

Following Vermersch’s initial take on how to analyse data collected from micro-phenomenological interviews (Vermersch, 2012), Petitmengin (2006) but also Valenzuela-Moguillansky and Vásquez-Rosati (2019) have refined and clearly specified the analytical process for research purpose.

Vermersch has proposed a process of repeated semiosis to gradually turn the interviewee’s experience into actionable micro-phenomenological knowledge. This process starts with the experience itself and how it is put into words by the interviewee. At this stage already, a semiotic transformation occurs, since some experiences are harder than others to express with words. The next stage is the transcription of recordings, during which various aspects of speech (pitch, intensity, rhythm) are often omitted for the sake of simplicity, although they may provide valuable information (it is, however, always possible to come back to early stages of the process of semiosis in search of useful information).

Below are details of our specific treatment of the transcriptions, with most steps commonly followed by researchers in micro-phenomenology: i) insert A and B in front of the uninterrupted interventions of the interviewee and the interviewer, respectively, and number the interventions one after the other for later referencing, i.e., B1, A2, B3, A4 etc.; ii) discard aborted sentences, repetitions/duplications, “uh”, indications of pauses or timing and brief interventions of agreement to increase readability (once again, all the steps of the progressive distillation of the initial material are preserved in case coming back to them would prove useful at a later stage of the analysis); iii) keep A’s descriptions of her/his experience and discard all other non-experiential pieces of information,, such as comments, opinions etc. Some key contextual elements can, however, be preserved if they shed meaningful light on the report of experience. For instance, interventions from B such as recapitulations can be deleted, but some key questions can remain if they usefully show how A’s attentional process is guided. Questions without which A’s answers would not make sense are recoded and appear with parentheses to prevent confusion with words actually uttered by A, e.g., [B: *“how did you feel?”* A: *“a bit nervous”*] becomes [A: *“(I feel/felt) a bit nervous”*]. Everything that is not experiential description is presented with a specific ink colour to clearly delineate it; iv) to ease understanding, move complementary information that appears later in the transcription to an earlier statement if it clarifies and/or specifies it (in connection with vii. below); v) underline verbs pointing to the interviewee’s activity—perceptions, sensations, mental activity, speech, actions etc. (e.g., *“I tell myself”, “I see that”, “I propose”, “I notice”, “I ask”* etc.)—to highlight the interviewee’s commonly favoured sensorial modalities and actions; vi) cut and organize the textual content in a succession of key moments, with corresponding labels, to build a comprehensive bird view of the lived experience; vii) further reorganize the content in order to fully shift from the chronology of the interview (V2) to the chronology of the experience itself (V1); viii) delete the numbering of the interventions for the sake of readability.

The result of the previous steps is called a “time unfolding” of the experience and is the basis for further analysis. In the specific case of the present study, in which A (the caregiver) is describing a first meeting with a patient, some actions of this patient, as perceived and reported by the caregiver, were kept to ease the understanding of the situation and of its dynamics. Also, some patients were accompanied, especially younger ones, and therefore the caregiver’s experience could also include this/these additional person(s).

While the early stages of the process of transformation of the transcriptions are rather straightforward, later stages, and especially stages vi and vii, often require efforts and are more interpretative. It is sometimes difficult to reconstruct the chronology of the experience with confidence as the interviewing process, with the fragmenting of the time of the experience, often disconnects and disseminates temporally contiguous micro-experiential events.

Beyond the previous process of extraction of actionable information, Vermersch also proposed a microscopic *model of action* (Faingold, 1997; Vermersch, 1994). It articulates several successive phases during action and is useful both as a support to guide the interviewee to describe her/his experience during the interview and to later organize it chronologically in a meaningful way. This model is reminiscent of earlier ones, which attempted to account for what took place at a cognitive level between a stimulus and a response, such as the TOTE (Test-Operate-Test-Exit) model (Miller et al., 1960).

As displayed in Figure 3, the process of action starts with 1) collecting information, then goes on with 2) identifying relevant information, 3) making a decision and 4) carrying out/implementing what has been decided. Interviewees usually report the second and fourth steps more spontaneously but can then be questioned explicitly on the other steps.

**Figure 1.**
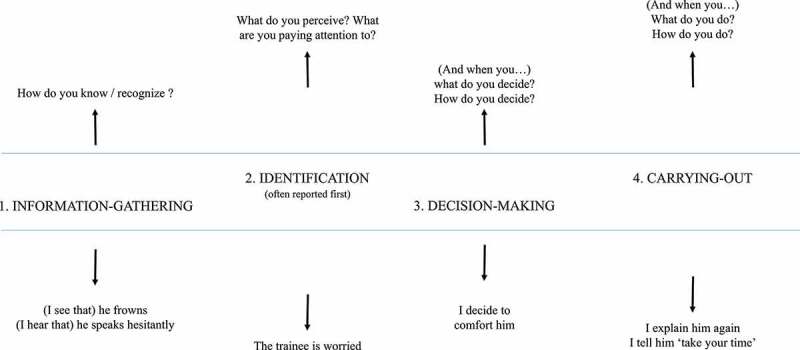
Description of the three main dimensions of micro-phenomenological phenomena: acts, objects of attention and experiential modalities.

**Figure 2.**
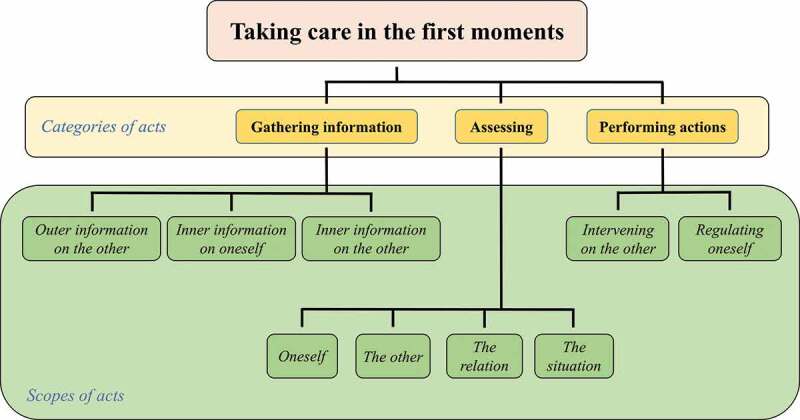
Instances of acts for the categories and scopes of acts.

**Figure 3.**
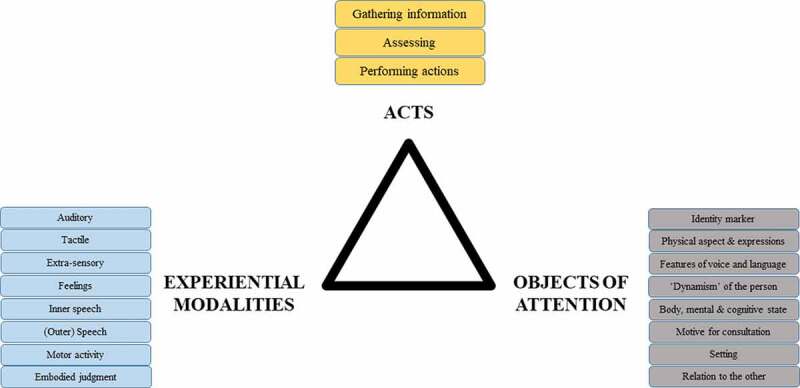
The model of action with related possible answers and questions in a micro-phenomenological interview.

Once the “time unfolding” of the experience has been reconstructed, one has access to a rich and condensed description, with the articulation of successive micro-experiential phenomena composed of perceptions and sensations, actions, decision-making etc. Such material was analysed to reach the results reported in the next section.

## Results

To better characterize the experience of meeting a new patient for the first time, we analysed in a qualitative way the micro-components of the caregivers’ experience, as extracted from the verbatim of our interviews and chronologically reorganized. We aimed to devise several complementary dimensions and, within each of them, a number of categories to reflect the diversity of situations and actions performed. We argue that characterizing the panorama of possible micro-experiences is the first step to a deeper understanding—without an accurate description, a phenomenon cannot be properly investigated.

From an epistemological point of view, our data could be analysed with either a bottom-up approach—extracting “emerging” descriptive categories from the verbatim—or a top-down one—projecting categories derived from theory on the verbatim to organize it. As we thought both perspectives had their benefits and limits, we chose to adopt an intermediate standpoint, taking advantage of well-identified concepts, especially from micro-phenomenology, while still staying faithful to our data when delineating a set of categories.

The extracts of the verbatim reported below are English translations of the French material. The sources of these translations can be found in Table I and Table II of the appendix “Original verbatim in French”.

**Table I.** Categories and scopes of acts performed by the caregiver appearing in the verbatim of the interviews, with illustrations

Category of acts	Scope of acts	Example of verbatim (translated to English)
**Gathering information**	Outer information on the other	*I see someone (a bit scared) who hastily puts back a magazine a comic book or a journal in the magazine rack on the coffee table who grabs her coat*
	Inner information on oneself	*There is like some heat in a corner of my head*
	Inner information on the other	*There is a resonance/it’s in my body/I feel there’s an emptiness where the other is*
**Assessing**	Oneself	*I tell myself that I’m not the person that people think I am at this moment*
	The other	*I find that the father and the son are a lot alike (laughs)*
	The relation	*I tell myself “ah” there’s going to be something dynamic I feel that the appointment is going to be dynamic (…) It’s something experienced by the whole body*
	The situation	*I tell myself that once again this is going to be a difficult story to hear*
**Performing actions**	Regulating (on oneself)	*I try to keep this reassuring attitude towards him while telling myself that it’s also like this that what comes afterwards will be of good quality*
	Intervening (on the other)	*By looking, I collect what he himself brings at that moment*
	Others	*I also start to squirm*

**Table II.** Experiential modalities for the caregiver with examples of verbatim

Experiential modality	Example of verbatim (translated to English)
**Visual**	*His gaze was alert his eyes bright and shining his gaze is not lifeless*
**Auditory**	*I perceived at that moment that he had a severe articulatory disorder*
**Tactile**	*I actually pay attention (…) to the handshake*
**Extra-sensory**	*He doesn’t exist (…) If I tried to touch him there would be no texture it would fall apart or it would be a ghost and I would pass through it it’s a feeling that the other person has no substance*
**Feelings**	*I feel that the arms relax and then I even feel that the shoulders relax there is an opening near the clavicles*
**Inner speech**	*I tell myself “ah” there’s going to be something dynamic*
**(Outer) Speech**	*I say ok I am ready to work with you about the rape*
**Motor activity**	*I am the one who opened the door*
**Embodied judgement**	*There is this slightly global idea of “ah” both of them are making a good impression*

### Main dimensions of experience

To guide us in the categorization of micro-experiential phenomena (MEP), we initially considered the two fundamental articulations of micro-phenomenology: i) the succession of these MEP through time, i.e., their diachrony, and ii) their various dimensions at any given moment, i.e., their synchrony.

At the diachronic level, we started from the model of action and its four causally related steps: i) gathering information, ii) identifying the situation (based on the information collected), iii) making a decision (after identifying the stakes of the situation) and iv) carrying out. We found that the third step was rarely identifiable as such in interviews, and was rather inferred from the fourth step onwards, as if it were in the “undercurrent” of the carrying out of action. We thus decided to drop it and consider three categories of acts—in the broad sense of the term, i.e., including perceptual acts, cognitive acts, speech acts, motor acts etc.—performed by the caregiver. We labelled these categories by adapting the terms of the model of action to better suit our verbatim, in order to remain close to the verbs most often used by the caregivers: i) “Gathering information”, ii) “Assessing” (rather than identification) and iii) “Performing actions” (as a simplification of the concept of carrying out a decision, since the process of decision-making often remained undescribed). We noticed in particular that assessments sometimes led to actions, but not always.

Considering the various synchronic dimensions of micro-experiences led us to further devise several scopes for the previous acts. They specifically reflect the situation of care, with the caregiver and the patient as main actors. They also characterize the caregiver’s perspective on this situation. For information gathering first, regarding the source of information, we first found that the caregiver gathers outer information, i.e., outside of her/his body, but also often inner information, i.e., within her/himself. In terms of scope, while one may initially associate outer information with the other—the patient—and inner information with oneself—here, the caregiver—, we found that inner information could also be related to the other. This highlights a possibly neglected source of information in at least some medical practices. We did not, however, find outer information about oneself, although this could have been possible, e.g., when one looks at her/his facial expressions in a mirror, or hears a recording of her/his own voice, i.e., elements not accessible “from within”.

**Figure 4.**
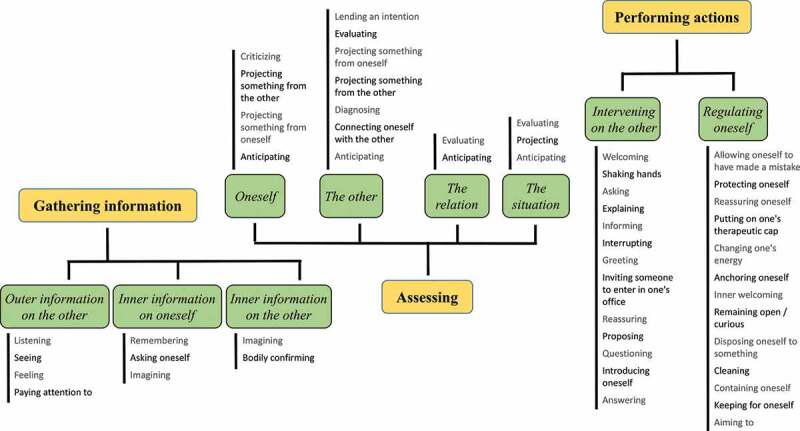
Organization of the acts performed by the caregiver during her/his first meeting with the patient in terms of categories and scopes of acts.

In a similar fashion, we observed that assessments could relate to a variety of scopes: the caregiver her/himself, the patient, but also their relationship or the situation, for instance, the perceived mood in the environment. Finally, in terms of actions, the caregiver could either regulate, i.e., act upon her/himself, “intervene”, i.e., act on the patient, or yet do something else. Table I summarizes our attempt and illustrates each possible situation with some verbatim. Figure 4 further explicitly connects categories and scopes of acts to the activity of taking care in the first moments of a meeting with a new patient.

g375

In addition to the previous categories and scopes of acts, we concluded to the need to specify the main experiential modality of either the gathering of information, the assessments or the actions of the caregiver. These modalities partly overlap with our five senses, but some of the latter are absent, while additional conditions were required. We thus considered:

- the visual, auditory, and tactile modalities from the five senses—there were no instances of olfaction or taste;
- the extra-sensory modality, which covers perceptual experiences which do not relate to any single perceptual modality but which are perceived other than with the organs of perception;
- feelings, i.e., perceptions which do not overlap with the five senses;
- inner speech, i.e., linguistic productions directed towards oneself;
- (outer) speech, directed towards others;
- motor activity;
- embodied judgements. This category refers to intellectual feelings, i.e., unanalysed impressions becoming ideas, judgements, and reasoning (Burloud, 1927). These conclusive, expert, and intuitive judgements usually come after a series of taking information and judgements, in the form of an insight. As Burloud writes, “the subject knows what to do but he has no representation of it”.

For instance, data collected outside of the body could be of a visual or auditory nature, internal information could consist in sensations, actions could consist in physical actions but also speech towards the patient or inner speech towards oneself. Table II provides illustrations for the various experiential modalities.

### Objects of attention and acts

The previous categories and scopes of acts are quite “high-level” and do not exhaust the content of our verbatim. More precise instances of acts performed by the caregiver but also objects of attention can be provided to get a better perspective of what the caregiver may experience. We delved into these elements as a mean to flesh out the previous main dimensions of experience. By doing so, various pieces of information, assessments, regulations, and interventions can be highlighted, which are meaningful to understand both the strategies implemented by the caregiver and her/his more spontaneous reactions in front of the patient.^4^ We paid close attention to the words used by interviewees to extract these finer-grained categories, hence their number.

Table III reports the categories of object of attention we identified and their members. They focus mostly on the patient and her/his observable features, but also cover unobservable body and mental states which are inferred to be hers/his. This is understandable given the broad objectives of caregivers during a first meeting. Besides, the latter also pay attention to their inner reactions to the patient, to elements which define and influence them such as their personal history, and to the relation they are building in a global and unfolding situation. These objects of attention offer an inner, intimate perspective on actual external phenomena; while the latter could be captured with an objective approach, only a subjective approach can reveal the former.

**Table III.** Objects of attention and their categories

Category of objects of attention	Objects of attention
**Identity marker**	First name and last name
**(Observable) physical aspect and expressions**	General appearance, hair, clothing & accessories, social class, gaze/eyes, smile, height, face
**(Observable) features of voice and language**	Coherence, elocution, loquacity, modulation, language register, expressions, voice (rate, pitch …)
**“Dynamism” of the person**	Attitude, dynamism, listening skills, intentional gestures, non-intentional gestures, handshake, way of walking, body posture, embodiment, breathing, silence, physical tension
**(Unobservable) body or mental state**	Abilities, internal states (emotions, feelings, mental readiness), psychic state (related to a pathology), internal images, thoughts, knowledge
**Motive for consultation**	Personal history, consultation request, personal issues/problems
**Setting**	Atmosphere (ambiance, energy), environment, situation, “framework”
**Relation to the other**	Welcome/reception, adjustment to the other, relational attitude, caregiver’s own body feelings, patient’s readiness for treatment, caregiver’s personal history, caregiver’s therapeutic posture, relational situation (in part. Therapeutic alliance)

Figure 1 summarizes and explicitly connects the different dimensions of micro-experiential phenomena, namely acts, experiential modalities and objects of attention.

In terms of precise instances of acts, Figure 2 reports a list for each scope under the categories of acts. These lists outline the diversity of acts rather than they offer an organization based on principles such as completeness, homogeneity of levels of description or mutual exclusivity. For instance, for “gathering outer information on the other”, one finds “listening”, “seeing” and “feeling” but also the more encompassing “paying attention to”. Among other interesting points, we can highlight that the act of “projecting something from someone to someone” occurs in various configurations under “Assessing”: from the other to oneself, from oneself to oneself (considering different states or ages), from the other to the other, and from oneself to the other. Additionally, the different acts under “Regulating”, which would be invisible to an external observer, reflect the multiplicity of strategies or reactions available to the caregiver. One can distinguish in particular several acts of kindness or benevolence towards oneself (“allowing oneself to have made a mistake”, “protecting oneself”, “reassuring oneself”) and towards the other (“remaining open/curious”, “inner welcoming”).

## Discussion

### Benefits of the micro-phenomenological interview

We argue that our approach offers some benefits compared to other common approaches, for instance, the phenomenographic one. Phenomenography focuses on lived experience just like we did with the micro-phenomenological interview, but how data on this experience are collected differs. The phenomenographic method relies essentially on the posture of the interviewer—more than on her/his technique. S/he must show great empathy towards the interviewee, introduces the topic of the interview with an open question, and then lets the interviewee speak, guiding her/him if necessary. This guidance is based essentially on the researcher’s interest in the participant’s experience and openness to the answers given.

In comparison, the micro-phenomenological interview relies on more precise technical choices when guiding the interviewee’s attention. Its key advantage is that it gives access to the pre-reflective experience, something the interviewee has difficulty exploring on her/his own but can access under the careful guidance of a trained interviewer. In particular, it is possible to access pre-reflective micro-actions such as information gathering and decision-making, which underlie identification and carrying-out, as we have presented in the model of action (cf., Figure 3). These micro-actions are very valuable to understand one’s activity because they correspond to the criteria on which s/he relies on to do what s/he does.

### Focusing on the invisible

Our contribution is an experiential study of the activity of taking care, in the first moments of a first caregiver-patient meeting. We centred our attention on the caregiver’s point of view, especially to highlight the importance of what she/he projects onto the patient within the clinical relationship.

It has been shown that countertransference—a constitutive process of the clinical relationship during which arise the caregiver’s projections, ideology, feelings, past experience, etc.—is as decisive as transfer from the patient in the psychotherapeutic relationship (Devereux, 1973). The projections that caregivers develop about their patients do not, however, only concern situations of psychotherapy. Indeed, our analyses show that these processes occur for all the caregivers we have interviewed. As such, they highlight a unifying aspect of care across different professions.

Beyond the previous point, in an attempt to lift the veil on the invisible side of caregivers’ practice, we studied their experience of situations of first meeting with patients so as to propose a dialectic between first-person cognitive science and practice, beyond the “cognitivist empire” (De Sousa Santos, 2018). Our analyses reveal inner practices among caregivers that play an important role, such as “gathering inner information”, either about oneself or the other, and performing different forms of regulation.

### Caregiving as a finalized activity

Like most professionals, caregivers work in the context of a finalized activity (Daniellou & Rabardel, 2005). A “finalized activity” is an “activity focused on the accomplishment of practical objectives” (Garfinkel, 1967) which differs from an “open-ended activity”, for which the expected results are indefinite or only loosely specified. For caregivers, among the main goals are the needs to establish a diagnosis and to build a therapeutic alliance (Rogers, 1957; Roth & Fonagy, 2006). We questioned them and they accordingly described actions that are relevant to making a diagnosis—for example, ‘*I tell myself at that moment, let’s go digging, maybe he’s not an autistic kid, maybe he’s high potential*’^5^ (a speech therapist) or “*there’s really this hat as a therapist that’s there that’s really there and that, hmm (taking a deep breath), knows*” (a psychotherapist). They also designated actions participating in the construction of a therapeutic alliance—“*and then I ask him to tell me who lives with them in the house. Once he has mentioned all the people in the family, I ask him if there are goldfish, turtles, hamsters other living people*” (a nurse).

We found in our data much verbatim related to the category “Gathering information”, which confirms that collecting information is at the core of caregivers’ activity. For instance, a speech therapist told us “*I say to myself, there’s going to be something dynamic, I hear that and I feel that the interview is going to be dynamic, at the same time as I say it to myself, I feel that it’s being experienced by my whole body*”.

The category “Assessing” that emerged from the inspection of our verbatim also relates to the notion of finalized activity. It articulates it, at least partly, with the model of action that occupies a central place in micro-phenomenology. More precisely, we have preferred “assessing” to the more general “identification”, since it corresponds more faithfully to the intention of making a diagnosis expressed by the caregivers themselves. For instance, a psychologist declared “*one sees there is suffering, discomfort … and it moves really fast her body and gestures change quickly as she evokes it*”, while a psychotherapist, using a diagnostic term, related that “*(…) meticulous, scrupulous, he has a very contrived way of doing things, I see that it’s a guy who has obsessive compulsive disorders*”. These descriptions illustrate how caregivers pre-reflectively take information in order to diagnose. The experiential and pre-reflective dimensions of the assessment of the situation thus seem quite fundamental constituents of the finalized activity of diagnosis. In particular, in our interviews, we identified some intellectual feelings, which turned out to be embodied expert assessments, which relate to and crystallize the caregiver’s whole expertise and the multitude of situations s/he has previously experienced professionally. For instance, during a session, while one of our caregivers gathers information concerning his patient’s attitude, appearance, and silence, he also does so about his own bodily sensations: he notices his altered breathing as well as a tension behind his neck and head. He makes several assessments about the “quality of silence” and the presence of his patient, and then comes to the following conclusive intellectual feeling: “*I feel something strange, I feel that something is happening to him which is a bit strange, I am telling myself there is something here which is not right*” (a psychotherapist). Of course, what he “feels” is not a feeling strictly speaking but rather a summative experience in the light of Burloud’s work.

### Caregiving as a productive and constructive finalized activity

Any finalized activity is simultaneously “productive” and “constructive”. It is productive since it is focused on the accomplishment of a project, according to the characteristics of the situation. It is also constructive since it is involved in the development of external and internal resources—such as instruments, skills, concepts or value systems (Rabardel, 2005). In relation to these two facets of finalized activity, experience has a dual nature. First, it is a “product” of activity, which to us connects well to the concept expressed by the German word *erlebnis*, i.e., the fact of having lived something, as well as the set of thoughts, perceptions, and sensations that this experience has aroused. Most verbatim illustrates the productive facet of the caregiving activity, for instance, when a dentist says “*I hold my breath, I breathe with my upper body, I prepare myself for everything to take longer with that person*”. Secondly, experience can be considered as a material in its own right, an object worked upon by constructive activity. While productivity relates to *erlebnis*, this evokes the concept of *erfahrung*, i.e., the experience one gets from doing something, often over a long period. An explicit illustration of how a caregivers’ knowledge manifests itself experientially is given by the example of a doctor who reported “*That’s where I start to look a little at semiology, like ‘how does he move?’, at analysing it. It’s more of a global situation, like a compilation of patients who had the same mode of movement, a sum of clinical cases*”. It is worth noticing that during micro-phenomenological interviews, the interviewee describes her/his specific subjective experience, lived at a particular moment, which corresponds to the *erlebnis*. But when s/he describes an experience that corresponds to personal knowledge, this also concerns the *erfahrung*, as it occurs in the *erlebnis*, e.g., “*I immediately think of technical observations. I make connections with the kids I’ve had to meet; I think of another patient I knew when she was the same age and with whom I loved to work*” (a speech therapist).

Some caregivers (psychotherapists, psychologists, etc.) are trained to stay aware during the session of all that is happening in the relationship: all that they notice with their patient but also all that they feel, think, experience in relation to their patient—which has been conceptualized under the term countertransference in psychoanalytically-inspired psychotherapies. Our study shows that similar projections onto patients do, in fact, occur within all caregivers. For instance, a nurse told us “*I feel like he wants to go and play, I feel like he’s not going to tell me much more*”, and a speech therapist “*I know that he very much wants this assessment*”. Indeed, unlike what happens in everyday life, the caregiver, ideally, tends to be reflexively conscious of what is affecting her/him in the relationship with the patient, so as to use this information to build her/his diagnosis. Therefore, understanding the productive facet of caregivers’ activity, in its experiential dimension, and the way it is articulated with its constructive facet, may be considered during caregivers’ training, mainly to give them keys to realize how they can build fruitful relationships with patients. For instance, light may be shed on the ways in which they can turn their attention to the atmosphere of a meeting with a new patient, the issue(s) possibly hidden behind the explicit motive for consultation, etc. We think that such considerations could help to shift from sometimes contrived and administratively constrained meetings to more qualitative and beneficial encounters.

### The healthcare system as a scene for caregiving

A phenomenology of taking care is to be framed in the context in which caregivers’ work and experience pressure to perform from their institutions. The environment of a first meeting does not suppress how socio-economic variables affect clinical practice in many countries today. Caregivers meet patients within a hyper-regulated health system, which is dominated by capitalistic principles and conditioned by the “tyranny of bureaucracy” (Dejours, 2007). This leaves little time to “truly encounter” a patient during a meeting.

The Covid-19 pandemic has exposed the deleterious effects of this system, and how caregivers often support others at the expense of their own wellbeing. In their phenomenographic study about the presence of health care professionals during their encounter with patients, Osterman and Schwartz-Barcott (1996) showed that they can be present in different ways, from being physically present but emotionally absent to being entirely present emotionally speaking and fully focused on the patient. The quality of presence of caregivers with their patients is negatively impacted by their stress level, which then affects the quality of care delivered. Such stress and feeling of being under pressure have increased since the beginning of the pandemic (Aledeh & Adam, 2020; Dullius et al., 2021; Morley et al., 2020). The interactions between caregivers and their patients are technocratic, and thereby routinized and strictly controlled, yet focused contextually on individual action. In the current pandemic context, on the one hand routines have been broken while, on the other hand, the workload has considerably increased, due to the large number of patients.

In our opinion, an answer to the previous issues, besides decreasing the overregulation and bureaucracy-induced stress, is to support caregivers through reflective practice so that they can express their sensations, feelings and thoughts about the care provided, beyond technical aspects and relational considerations. This is necessary both for the maintenance of a certain quality of care and for the caregivers’ own health. Importantly, it can be facilitated using analytical data from an experiential approach as we have proposed. Knowledge arising from experiential research indeed enhances a holistic understanding of caregivers’ practice and expertise. It can show in particular how the finesse of this expertise is actually nestled in the folds of their pre-reflective activity. Highlighting this important aspect carries implications for future training in clinical practice and suggests finding more opportunities and space for caregivers to contact with their lived experience and learn from its multiple facets. Becoming more aware of how and why one reacts in front of a patient, understanding retrospectively why a moment decision was taken or a quick judgement was made, can help to fine-tune one’s behaviour and expertise. In short, reflectivity can enhance reflexivity. Beyond that, it may also assuage a caregiver’s need to find out who s/he truly is in front of a patient.

## Conclusion

We have tried in this paper to better understand what is at play in caregivers’ daily practices with their patients, in particular when it comes to the establishment of both a diagnosis and a therapeutic alliance—which are at the core of the finalized activity of caregiving. Relying on micro-phenomenology and the explicitation interview, we have especially investigated the subjective experience of taking care of someone else in the first few moments of the first meeting. We have highlighted how the caregiver-patient relationship is structured experientially, notably via micro-experiential phenomena that we have described at the diachronic and synchronic levels. We have attempted to organize these phenomena with categories of acts, scopes of acts, and experiential modalities. At a finer-grained level, we have identified a number of objects of attention and acts. What emerges from this approach is the importance of gathering not only outer but also inner information about the patient, of the multiple factors involved in assessing this patient’s case besides direct observation and listening, and of silently regulating one’s activity. The pre-reflective mode of consciousness plays here an important role, which can only be revealed with a first-person approach. In front of the challenges faced by caregivers, these results suggest developing a reflexive practice which includes what the reflective study of lived experience has to offer.

## Endnotes

1. We follow Vermersch (2017), who distinguishes between reflection and reflexivity: reflection is the first step of awareness according to Piaget (1977). It is the transformation of an experience, which was only lived, into a representation. Reflexivity, on the other hand, means using the lived experience as an object of knowledge after it has been represented.

2. For us, and according to Depraz et al. (2003, p. 2), “experience is always that which a singular subject is subjected to at any given time and place, that to which s/he has access ‘in the first person’”.

3. We adopt Petitmengin (2007)’s definition of pre-reflective dimension when she writes (p. 55) “We use the term ‘pre-reflective’ in order to emphasize the fact that this dimension is not unconscious, but only not yet conscious”.

4. The distinction between deliberate strategies and spontaneous reactions is over-simplistic. A continuum along various degrees of intention would be a more sensible representation.

5. In this verbatim as well as in the following ones, we have added punctuation for the sake of readability. We are aware that this differs from standard rules of transcription, which we have followed with our recordings.

## Appendix: Original verbatim in French

**Table I.** Categories and scopes of acts performed by the caregiver appearing in the verbatim of the interviews, with corresponding illustrations in French and their English translation

*Category of acts*	*Scope of acts*	*Example of verbatim* *(in French)*	*Example of verbatim (translated to English)*
**Gathering information**	Outer Information on the other	*j’vois quelqu’un (un peu effrayé) qui repose précipitamment un magazine une bd ou une revue sur le porte-revues sur la table basse qui attrape son manteau*	*I see someone (a bit scared) who hastily puts back a magazine a comic book or a journal in the magazine rack on the coffee table who grabs her coat*
	Inner Information on oneself	*y a comme une chaleur dans un coin de ma tête*	*There is like some heat in a corner of my head*
	Inner Information on the other	*il y a une résonance/c’est dans mon corps/je sens qu’il y a un vide là où est l’autre*	*There is a resonance/it’s in my body/I feel there’s an emptiness where the other is*
**Assessing**	Oneself	*j’me dis que j’suis pas la personne que les gens pensent que j’suis à ce moment-là*	*I tell myself that I’m not the person that people think I am at this moment*
	The other	*je trouve que le père et le fils se ressemblent beaucoup (rires)*	*I find that the father and the son are a lot alike (laughs)*
	The relation	*je me dis tiens là il va y avoir quelque chose de dynamique je sens que l’entretien va être dynamique (…) c’est vécu par tout le corps*	*I tell myself “ah” there’s going to be something dynamic I feel that the appointment is going to be dynamic (…) It’s something experienced by the whole body*
	The situation	*je me dis que ça va être encore une histoire difficile à entendre*	*I tell myself that once again this is going to be a difficult story to hear*
**Performing actions**	Regulations (on oneself)	*j’essaie de garder cette attitude rassurante à son égard en me disant que c’est aussi comme ça que ce qui va suivre sera de bonne qualité*	*I try to keep this reassuring attitude towards him while telling myself that it’s also like this that what come afterwards will be of good quality*
	Interventions (on the other)	*avec le regard je vais chercher ce qu’il amène lui à ce moment-là*	*By looking, I collect what he himself brings at that moment*
	Others	*je me mets aussi à me tortiller*	*I also start to squirm*

**Table II.** Experiential modalities for the caregiver with corresponding examples of verbatim in French and their English translation

*Experiential modality*	*Example of verbatim* *(in French)*	*Example of verbatim* *(translated to English)*
**Visual**	*il avait le regard assez réveillé des yeux clairs assez lumineux il n’a pas le regard éteint*	*His gaze was alert his eyes bright and shining his gaze is not lifeless*
**Auditory**	*j’ai perçu à ce moment-là qu’il a un gros trouble d’articulation*	*I perceived at that moment that he had a severe articulatory disorder*
**Tactile**	*je prête attention (…) à la poignée de main en fait*	*I actually pay attention (…) to the handshake*
**Extra-sensory**	*il n’existe pas (…) si j’essayais de le toucher il n’y aurait pas de texture ça s’effondrerait ou ça serait un fantôme je passerais à travers c’est une sensation que l’autre n’a pas de substance*	*He doesn’t exist (…) If I tried to touch him there would be no texture it would fall apart or it would be a ghost and I would pass through it it’s a feeling that the other person has no substance*
**Feelings**	*je sens que il y a une détente des bras et là je sens même qu’il y a une détente au niveau des épaules une ouverture près des clavicules*	*I feel that the arms relax and then I even feel that the shoulders relax there is an opening near the clavicles*
**Inner speech**	*je me dis tiens là il va y avoir quelque chose de dynamique*	*I tell myself “ah” there’s going to be something dynamic*
**(Outer) Speech**	*je dis ok moi je suis prête pour travailler avec vous sur le viol*	*I say ok I am ready to work with you about the rape*
**Motor activity**	*c’est moi qui ai ouvert la porte*	*I am the one who opened the door*
**Embodied judgement**	*il y a cette idée un petit peu globale de ah tiens ils présentent bien tous les deux*	*There is this slightly global idea of “ah” both of them are making a good impression*
